# Retraction Note: Human Umbilical Cord Mesenchymal Stem Cells-Derived Exosomal MicroRNA-18b-3p Inhibits the Occurrence of Preeclampsia by Targeting LEP

**DOI:** 10.1186/s11671-022-03765-6

**Published:** 2022-12-09

**Authors:** Qin Huang, Meng Gong, Tuantuan Tan, Yunong Lin, Yan Bao, Cuifang Fan

**Affiliations:** 1 Department of Obstetrics and Gynecology, Renmin Hospital of Wuhan University, 238 Jiefang Road, Wuchang District, Wuhan, 430060 Hubei China; 2 Ultrasound Imaging Department, Renmin Hospital of Wuhan University, Wuhan, 430060 Hubei China; 3 Department of Statistics, UW-Madison, Madison, 53703 USA

**Retraction Note to: Nanoscale Research Letters (2021) 16:27** 10.1186/s11671-021-03475-5

The Editors in Chief have retracted this article. After publication, concerns were raised concerning the appearance of Western blots. The authors did not respond to requests for further clarification and did not supply raw data or the ethics permit. The editors, therefore, have lost confidence in the integrity of the article’s findings. The editor was not able to obtain a current email address for Author Yan Bao. Authors have not responded to any correspondence from the editor or publisher about this retraction.

